# Climate-driven habitat shifts of high-ranked prey species structure Late Upper Paleolithic hunting

**DOI:** 10.1038/s41598-023-31085-x

**Published:** 2023-03-14

**Authors:** Peter M. Yaworsky, Shumon T. Hussain, Felix Riede

**Affiliations:** 1 Department of Archeology and Heritage Studies, School of Culture and Society, Aarhus University, Moesgård Allé 20, Building 4216, 8270 Højbjerg, Denmark; 2 Center for Biodiversity Dynamics in a Changing World, Department of Biology, Aarhus University, Ny Munkegade 114-116, 8000 Aarhus C, Denmark

**Keywords:** Behavioural ecology, Palaeoclimate, Palaeoecology, Climate-change adaptation, Climate-change impacts

## Abstract

Changing climates in the past affected both human and faunal population distributions, thereby structuring human diets, demography, and cultural evolution. Yet, separating the effects of climate-driven and human-induced changes in prey species abundances remains challenging, particularly during the Late Upper Paleolithic, a period marked by rapid climate change and marked ecosystem transformation. To disentangle the effects of climate and hunter-gatherer populations on animal prey species during the period, we synthesize disparate paleoclimate records, zooarchaeological data, and archaeological data using ecological methods and theory to test to what extent climate and anthropogenic impacts drove broad changes in human subsistence observed in the Late Upper Paleolithic zooarchaeological records. We find that the observed changes in faunal assemblages during the European Late Upper Paleolithic are consistent with climate-driven animal habitat shifts impacting the natural abundances of high-ranked prey species on the landscape rather than human-induced resource depression. The study has important implications for understanding how past climate change impacted and structured the diet and demography of human populations and can serve as a baseline for considerations of resilience and adaptation in the present.

## Introduction

The difficulty of disentangling the interrelated effects of climate impacts and population pressures on the archaeological record has long been recognized^1–9^. The issue is particularly crucial for understanding subsistence shifts reflected in the zooarchaeological record^5,8–12^: Are changes in species compositions of the zooarchaeological record primarily a product of: (i) changing environments altering species’ natural abundances in those environments, (ii) human populations depleting high-ranked resources resulting in a broadening of the diet leading to the incorporation of lower-ranked resources, or (iii) a combination of the two? Archaeological research has produced a rich body of literature structured around two contrasting positions, one foregrounding climate-driven explanations for changes in faunal representations^13–17^, the other advocating in favor of overhunting and anthropogenic impacts^1,18–21^, and in rare cases, researchers have attempted to separate which factor is decisive for specific species^22^.

To address the problem, we synthesize spatiotemporal paleoclimate, archaeological, and zooarchaeological data using a combination of ecological theory and methods, specifically those derived from eco-informatics and human behavioral ecology^23,24^, to test the extent to which climate and human populations affect changes in subsistence observed in the Late Upper Paleolithic (LUP) zooarchaeological record. The framework implemented here allows us to: (i) identify high-ranked prey species for LUP foragers, (ii) estimate the distribution and relative abundances of prey species across Europe, and (iii) overcome issues of nonindependence between climate and human population estimates.

At present, we understand that humans have significant effects on contemporary and historical ecologies and animal populations^25–28^, but not how far back such impacts extend in time nor if there is an identifiable point in human prehistory at which human populations began having large-scale effects on mammalian biodiversity. Notably, the Pleistocene-Holocene transition has become an important candidate timeframe for such effects in recent years^9,29–32^. Moreover, the question of how humans responded to changes in the environment, whether they be human-induced and/or climate-driven, is of particular relevance in light of ongoing debates on global anthropogenic climate change in the present and future^33–35^.

There is a positive correlation between hunter-gatherer population size and warming temperatures^15,36,37^, with climatic variables having differential effects on hunter-gatherer populations^38^, and recent work showing a relatively narrow climate niche of Holocene *Homo sapiens*^35^. During the Terminal Pleistocene, the correlation between population and climate variables clouds our ability to deduce causation due to a number of interconnected events of interest, such as faunal extinctions, population collapse and exchange, subsistence shifts, and the species composition of zooarchaeological assemblages^1,5,6,13–15,18,19,22,39,40^. The difficulty of discerning whether human populations or climate are accountable for changing faunal compositions and frequencies observed archaeologically—and which critically inform understandings of past human ecology, adaptation and strategic decision-making—rests in the nonindependence of these variables, particularly for the Terminal Pleistocene where climatic but also faunistic changes were pronounced^41,42^.

**Figure 1:**
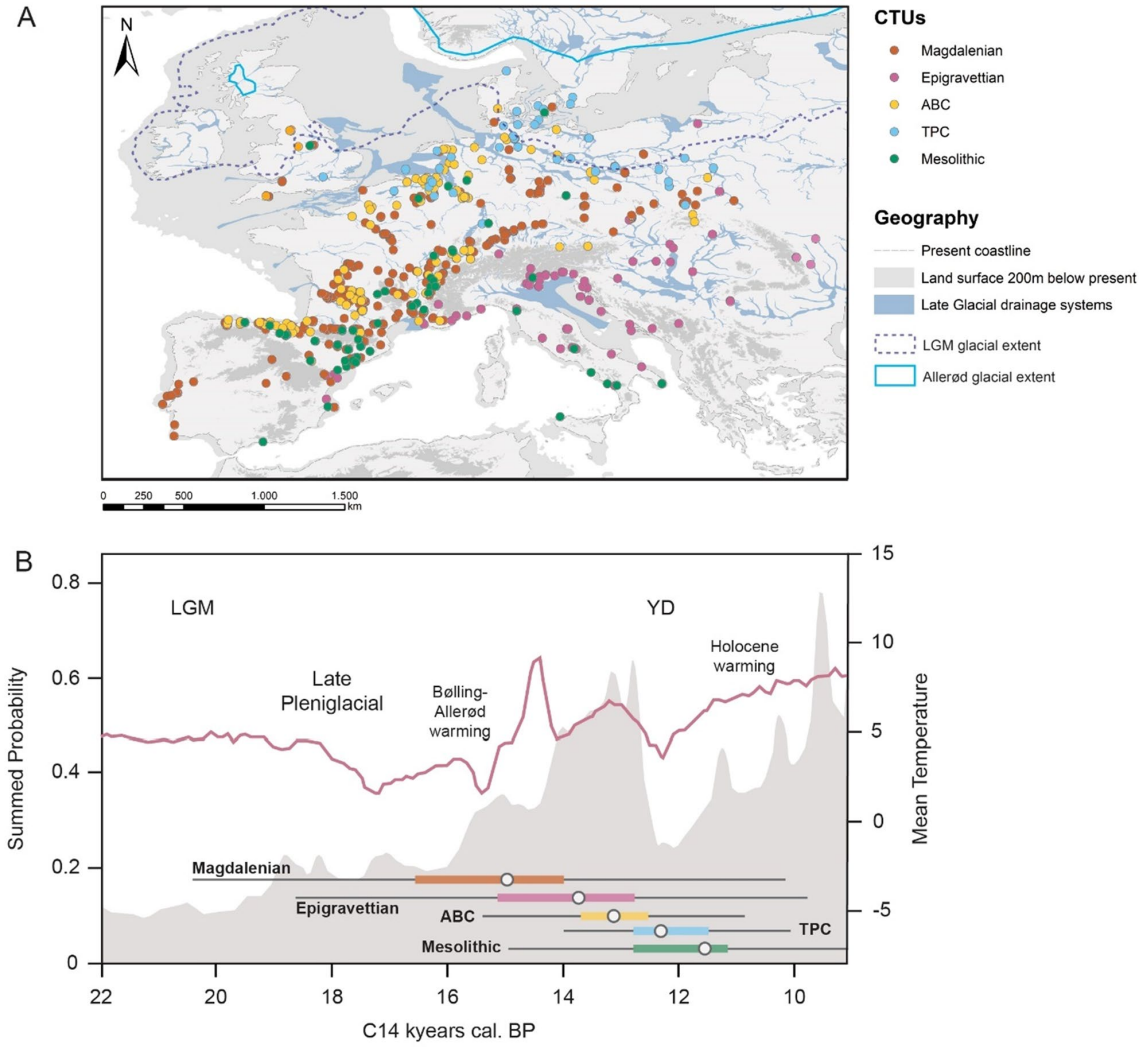
Geographic and chronological scope of the employed dataset. Commensurate with the large-scale, long-term pan-European approach adopted here, the archaeological data on site location, chronological position and zooarchaeological assemblage composition are aggregated under their associated higher-order Cultural Techno-Units (CTUs), yielding six archaeological macro-complexes: the Magdalenian, Epigravettian, Arch-Backed Complex (ABC), Tanged Point Complex (TPC), and early Mesolithic. (**A**) shows the spatial distribution of all analyzed sites and their CTU affiliation. (**B**) shows the change in mean terrestrial temperature in Europe from 22 to 9kya, with an SPD used to estimate changes in regional population density represented by the shaded grey area using calibrated radiocarbon dates drawn from the European subset of the P3K14C database. The color-coded bars present the median measured age and the distribution of all dates recorded for each CTU. The map was created in ArcMap 10.3.1 (www.arcgis.com). European GIS-elevation data is based on the SRTM open-source data for Eurasia provided by NASA/JPL^59^. Coastline, glacial extent and Late Glacial drainage system data are taken from the European prehistoric and historic atlas (EPHA) hosted by the Centre for Baltic and Scandinavian archaeology (ZSBA) in Schleswig, Germany (CC-BY 4.0 license)^55^.

The LUP in Europe (22–9 kya) overlaps with the end of the Pleistocene and the beginning of the Holocene, thus including the Final Paleolithic and the early Mesolithic. The period is characterized by rapid changes in climates^43–46^ and non-analog ecologies^16,47–53^. Changing climates affected both human and faunal distributions, thereby structuring human diets, population size, and cultural processes^36^. The period between the Last Glacial Maximum (LGM; ca. 25–22 kya) and the beginning of the Holocene (ca. 11.8 kya) is generally characterized by glacial recession and the opening of northern latitude landscapes for human foragers^54,55^. Traditionally, this expansion of human populations into Europe’s northern latitudes at the margins of former glaciers has been linked to a fragmentation of societies with regionally distinct economic and cultural expressions^56–58^ (Fig. 1). With its relatively well-resolved archaeological record, high temporal control, and robustly inferred human population growth trajectories, climate change patterns, as well as faunal turnovers and habitat shifts, the period represents a unique window into early human prehistory for disentangling the interrelationships between contemporaneous climate, human populations, and their prey species.

To address the methodological concerns and answer the question of causality in zooarchaeological assemblage composition change, we implement the following analytical framework:

1. To identify the high-ranked prey species during the study period in Europe, we revisit and update post-encounter return rate estimates derived from the generalized Prey Choice Model (PCM^60–62^) with estimates of pursuit failure.
2. We then estimate past distributions and relative abundances of the top-ranked species through time by creating species-specific spatiotemporal species distribution models (SDMs) over downscaled centennial-scale and spatially explicit paleoclimate data^63^.
3. Using the archaeological data, we then use the date and location to extract the relevant spatiotemporal variables of temperature and suitability for the different species, thus incorporating the spatial and temporal variation from across Europe during the time.
4. We create a summed probability distribution (SPD) as a proxy for a regional population estimate from the comprehensive radiocarbon database P3K14C^64^.
5. We combine and extend independent archaeological^65–67^ and zooarchaeological databases^68^ containing information on site locations, cultural attribution and proportional zooarchaeological abundances (see “Materials and methods”).
6. Mobilizing these datasets, we use a piecewise structural equation model (pSEM) to discern the relative contribution of climate and population to changes observed in zooarchaeological assemblages during the LUP, while controlling for climatic change.

The pSEM^69^ we build allows us to determine the individual effects of the two variables on recorded zooarchaeological abundances of prey species throughout the LUP while accounting for potential *nonindependence* of climatic and human variables. Archaeological data on geographic site location, temporal position, and faunal assemblage composition are aggregated into higher-order Cultural Techno-Units (CTUs) serving as primary units of analysis to discern trends and specific inter-variable relationships. These CTUs allow us to comparatively assess the interplay between changing human contexts including foraging patterns, climates, and animal species distributions^70–72^, to chart shifting causal relationships at the human–environment interface. Our findings suggest that changes in zooarchaeological assemblage configurations in LUP Europe are primarily a result of climate-driven habitat shifts of key high-ranked prey species rather than anthropogenic resource depletion.

## Post-encounter return rate and pursuit failure

To begin to understand human foraging decisions we must determine the prey species most important to LUP hunter-gatherers’ subsistence, and thus high-ranked. To understand which prey species were high ranked, we (i) identify what mammals were generally present in the ecosystems of the study period, (ii) estimate their (post-encounter) profitability, and (iii) rank them according to their profitability.

Profitability is defined here within the PCM, as *post-encounter return rate* (PERR;^60^). PERR is commonly defined as energy divided by handling costs (measured in time) to produce a rate of energy acquisition (LHS of Eq. (1) in “Materials and methods”). Ranking prey species can be difficult, and in the past researchers have simply assumed that the mean body weight of a species corresponds with its ranking since body weight should roughly equal hunting returns^9,73–76^. While this may be true for some animal species, especially smaller animals with high pursuit success and low handling costs, the approach is demonstrably not appropriate for larger animals as the relationship between body weight and profitability is non-linear^77–81^. To derive a more robust measure for PERR and prey rank for mobile game we need to consider the increased handling costs of larger game and the probability of a failed pursuit.

To incorporate the probability of pursuit failure into PERR, it is vital to i) distinguish post-encounter pre-acquisition costs (e.g., pursuit costs) from post-acquisition costs (e.g., butchering, smoking, cooking, etc.), and ii) discount the post-acquisition costs and energy gained by the probability of pursuit failure (Eq. (2) in “Materials and methods”). The result is an elaboration on standard PCM PERR that overcomes over- and underestimating profitability issues present in previous implementations incorporating pursuit failure^78,81^. By incorporating pursuit failure into our determination of PERR, a forager’s decision to pursue a prey item does not necessarily result in acquisition of that prey item. This probability of failure then feedbacks into the forager’s assessment of the profitability of that prey item, and structures whether that resource is within the diet breadth and should be pursued upon encounter.

## Predictions

Predictions from PCM informs our piecewise structural equation models (pSEM). In this way, we obtain three empirical predictions that can be tested against the archaeological record and the model outputs of the pSEM:

1. High-ranked prey species should *always* be pursued upon encounter, regardless of their abundance^62^; their abundance in the archaeological record is therefore a measure of their abundance on the wider landscape. Higher-ranked species will consequently have better model fits in our analysis.
2. When the natural abundance of high-ranked prey species is greater on the landscape relative to other low-ranked species, we expect the high-ranked species to make up the majority of the zooarchaeological record, at least if their abundances are not substantially impacted by human hunting.
3. As high-ranked prey species decline in abundance (either from human hunting pressure or habitat shifts resulting from climate change) foragers should incorporate more lower-ranked species according to prey rank order, thus increasing their abundance in the zooarchaeological record. Yet the incorporation of lower-ranked prey items (diet breadth expansion) does not necessarily imply human resource depression but could also reflect changes in natural abundances of high-ranked pretty species due to habitat shifts and climate change.

The fundamental rationale of this PCM approach is that LUP foraging decisions are not directly reflected in the zooarchaeological record but are mediated by cost–benefit structures and the relative economic significance of different prey species. The same cost–benefit patterns of decision making are present throughout the LUP with differences in zooarchaeological assemblages stemming from differences between spatially and temporally distinct habitats. Both empirical convergence with and divergence from these expectations yield important insights on human–environment interactions in the period between the LGM and the Holocene.

## Results

### Prey ranking and post-encounter rates

Using African fauna post-encounter return data^79^, we estimate PERR using Eq. (2) (see Supplementary Information 1)^82^ for European species^6^. Excluding carnivores, the seven prey species with the highest PERR are, in order of decreasing importance, reindeer (*Rangifer tarandus*), boar (*Sus scrofa*), fallow deer (*Dama dama*), red deer (*Cervus elaphus*), ass (*Equus hydruntinus*), elk (*Alces alces*), and horse (*Equus ferus*) (Table 1).

**Table 1:** Prey rank of the animal species used in the analysis, along with their body weight and calculated post-encounter return rate (PERR).

Rank	Species name	Common name	PERR (cals/h)	Weight (kg)
1	*Rangifer tarandus*	Reindeer	4287	86
2	*Sus scrofa*	Boar	3926	117
3	*Dama dama*	Fallow deer	3683	65
4	*Cervus elaphus*	Red deer	3511	187
5	*Equus hydruntinus*	Ass	3370	230
6	*Alces alces*	Elk	3108	385
7	*Equus ferus*	Horse	3010	500
8	*Bison priscus*	Bison	2914	687
9	*Saiga tatarica*	Saiga	2893	29
10	*Bos primigenius*	Aurochs	2811	1050
11	*Capreolus capreolus*	Roe deer	2757	23
12	*Coelodonta antiquitatis*	Woolly rhinoceros	2583	2685
13	*Mammuthus primigenius*	Mammoth	2380	4285

Note that, as expected, body weight is not a reliable predictor for PERR as animal species such as aurochs, rhino, and mammoth rank tenth or lower when we consider their handling costs and probability of pursuit failure.

In the analyses that follow we focus primarily on five of the top seven prey items listed above excluding fallow deer and ass. In the study period, ass is only represented in paleontological databases, making modeling its paleo-distribution difficult and beyond the scope of this paper. Fallow deer are not present in Europe during the LUP. For the purpose of the pSEM and the detailed analysis of LUP prey choices, we moreover concentrate on the top three species returned from the remaining list of ranked prey items: reindeer, boar and red deer.

### Species’ habitat suitability and faunal composition by CTU

We combine the archaeological, zooarchaeological, population estimate, and habitat suitability datasets based on their shared CTU, distilling the disparate data into estimates of average species availability (based on the SDM results) and average zooarchaeological composition (See Table 1 in Supplementary Information 2)^82^.

**Figure 2:**
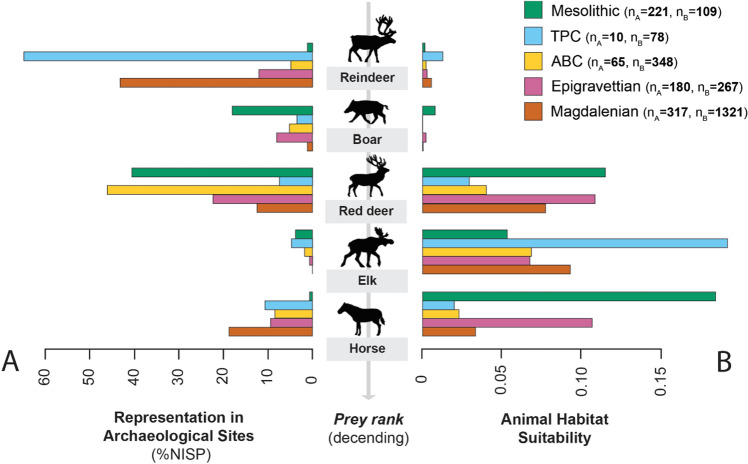
Comparison of zooarchaeological NISP proportions and climate-inferred habitat suitability for the top-ranked prey species. (**A**) presents the averaged zooarchaeological assemblage compositions attributable to each species for each CTU. (**B**) shows the reconstructed average habitat suitability for each species of each CTU. Each bar represents the zooarchaeological abundance (**A**) and the habitat suitability (**B**) for the corresponding top-ranked prey species colored by CTU affiliation. Note that these bars are not directly comparable between species. In comparing (**A**) and (**B**), it is evident that LUP foragers were not targeting species based on which species are most abundant, but on the species’ prey rank, with a preference for higher-ranked prey species.

Using the combined dataset, we can compare the habitat envelope of individual CTUs with regard to prey species suitability and the average composition of associated zooarchaeological assemblages to confront our theory-derived predictions (Fig. 2). There are notable differences between the analyzed CTUs both in terms of their animal habitat suitability profiles and the averaged relative zooarchaeological frequency of high-ranked prey species within them. The Magdalenian and especially the Tanged Point Complex (TPC) focus disproportionally on reindeer despite their habitats being suitable for other lower-ranked species, notably elk and red deer. In fact, reindeer is zooarchaeologically represented in all CTUs even when suitability scores are extremely low such as in the Mesolithic and this is consistent with PCM predictions. The same is true for boar, which is consistently taken by LUP hunter-gatherers, even when boar habitat suitability is low as in the TPC, the Arch-backed Complex (ABC) and the Magdalenian. This similarly suggests that prey ranking mediates foraging patterns. In the Mesolithic the pattern shifts as boar becomes abundant in the zooarchaeological record, paralleled by a marked increase in boar habitat suitability. Elk and horse are never the dominant species in the archaeological record even in CTU contexts in which their habitat suitability scores are elevated, reflecting their low prey ranking. The pattern for red deer is interesting and may reflect its intermediate ranking in the diet breadth of LUP hunter-gatherers. Red deer is the most frequent prey species in the averaged faunal samples from the Epigravettian, ABC and Mesolithic and this might reflect their abundances in the landscape as well as a possible inclination toward broader-spectrum diets in these CTUs, which are also all associated with a notable woodland component in their environments^56,83–85^. Taken together, this larger pattern illustrates the trade-offs between habitat suitability, prey abundances, and species profitability.

### pSEM: climate-population dynamics

**Figure 3:**
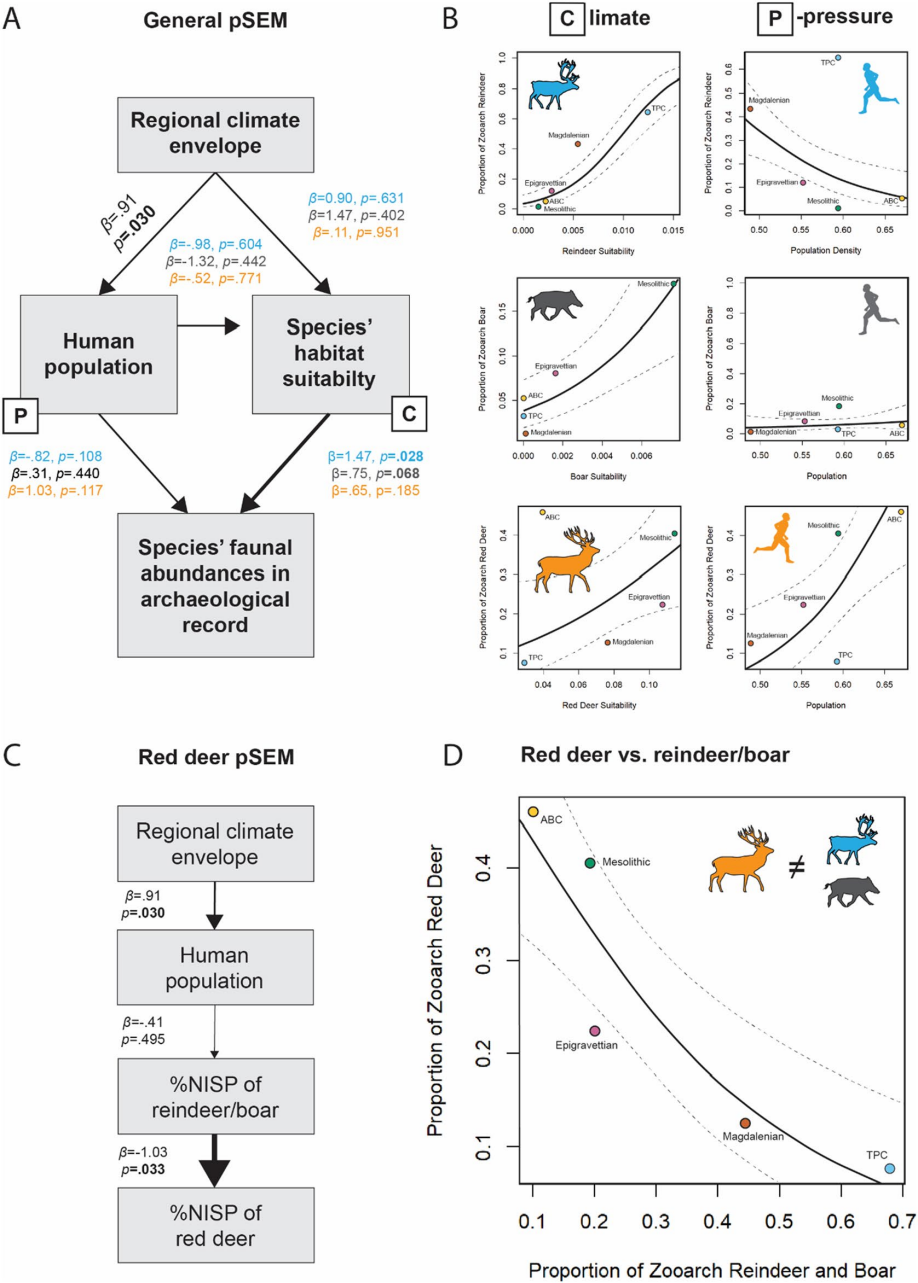
Relationship between climate and human population during the LUP based on the piecewise structural equation model (pSEM). (**A**) describes the structure of the pSEM used, wherein the zooarchaeological record is considered the product of the natural abundance of animal prey species in an occupied habitat and the effects of human population pressures on the available prey resources. The natural abundances of a species in an occupied habitat are then further influenced by human population densities and both are affected by regional climatic trends. (**B**) shows the model outputs for climate (C) and population (P) for each of the three considered top-ranked prey species (reindeer, boar, and red deer). (**C**) outlines the structure of the red deer-specific model, using an pSEM in which the abundance of red deer in the zooarchaeological record is structured by the abundance of reindeer *and* boar in the zooarchaeological record, which is regarded to be a product of human population density, in turn considered to be a product of regional climate. (**D**) shows the results of the red deer model.

Figure 3A outlines the structure of the piecewise structural equation model (pSEM) we use to address issues of non-independence between the main predictor variables. The model fits for each separate inter-variable relationship for the three top-ranked prey species (reindeer, boar, and red deer) are given in Fig. 3A. In all analyzed cases, human population density (P) correlates strongly with regional average summer temperature highs (C) (*β* = 0.91, *p* = 0.03). Other statistically significant relationships are highlighted in bold and pertain to the link between species habitat suitability and zooarchaeological abundance in the case of—as expected following the PCM—reindeer (*β* = 1.47, *p* = 0.028) and boar (*β* = 0.75, *p* = 0.068). The comparison between LUP populations and climate-driven species habitat suitability as predictor variables for the observed faunal abundances in the zooarchaeological record reveals that population (P) has a negligible effect on the observed patterns relative to climate (C).

Figure 3B shows the individual results for each animal-CTU pairing regarding climate (C) and human population (P) as explanatory variables. Consistent with the observations on the relation between CTU, habitat suitability, and zooarchaeological frequencies, climate is a good predictor for reindeer (*β* = 1.47, *p* = 0.028) and boar (*β* = 0.75, *p* = 0.068). The pSEM performance is considerably weaker regarding the link between climate and red deer hunting (*β* = 0.65, *p* = 0.185) and this may suggest that red deer is a strategic compromise or a second-choice target but occurs in appreciable abundance in most environments. The individual species-specific results of the effects of LUP population density yield a different pattern. There is no relationship for boar (*β* = 0.31, *p* = 0.44), reindeer (*β* = − 0.82, *p* = 0.108), or red deer (*β* = 1.03, *p* = 0.117).

### pSEM: red deer

The abundance of red deer within the zooarchaeological record is not strongly driven by the species’ habitat suitability (*β* = 0.65, *p* = 0.185) nor human population density (*β* = 1.03, *p* = 0.117; cf. Figure 3A,B). Since we expect lower-ranked prey items to be incorporated into the diet as the habitat suitability and relative natural abundance of higher-ranked resources decreases, we can test for this effect by using a slightly adjusted pSEM (Fig. 3C). Interestingly, we find that as the proportion of the zooarchaeological record attributable to reindeer *and* boar (the two highest-ranked prey species) declines, there is a significant increase in the proportion of red deer observed in the zooarchaeological record (Fig. 3D; *β* = − 1.0255, *p* = 0.033), and that while climate generally influences human populations, human population density does not seem to have an effect on the abundance of the combination of boar and reindeer (*β* = 0.495, *p* = 0.41). This strongly suggests red deer were hunted in habitats where higher-ranked resources (reindeer and boar) were less abundant, but *not* because of human hunting pressure on these two high-ranked prey species.

**Figure 4:**
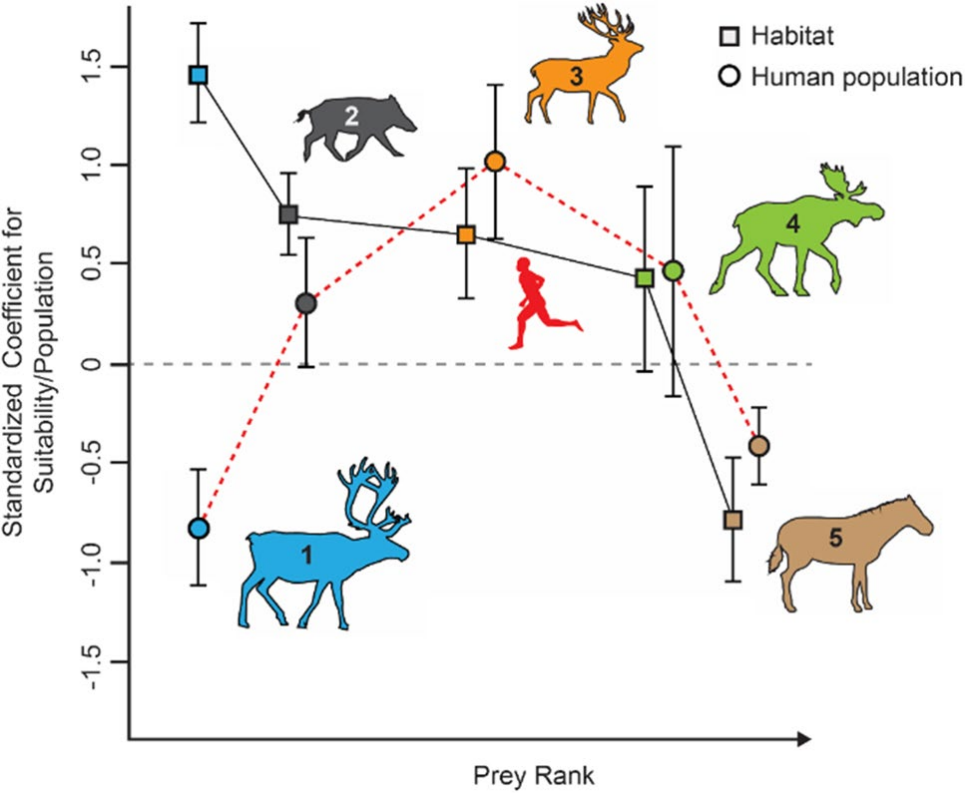
Relationship between top-ranked prey species habitat suitability and LUP human population impacts measured by standardized beta coefficients and standard error estimates for each individual species-specific pSEM. Squares represent the calculated coefficients for species habitat suitability. Theory predicts that as we descend in prey rank beta coefficients decrease while the error increases suggesting that models are performing worse the lower the prey rank, in turn suggesting a positive relationship between predictive strength and prey rank. Circles represent the coefficients for human population. There is a clear parabolic effect but note that the coefficients alone suggest zooarchaeological abundances of reindeer decline as populations expand and zooarchaeological abundances of red deer increase as population increase, although these relationships are non-significant in our models. The remaining values yield standard errors overlapping with 0.

Figure 4 presents the standardized beta values for all considered prey species. We can use these values to qualitatively examine whether species habitat suitability and human population density produce the kind of patterns predicted by PCM. Since the incorporation of lower-ranked resources such as red deer, horse and elk into LUP diets is not only structured by the natural abundances of these resources and the strength of human impacts, but also by the abundance of higher-ranked resources, we expect that the standardized beta values for habitat suitability will reflect this, with weaker values corresponding to lower prey ranks. The data follows this expected pattern. Higher-ranked prey items have stronger positive standardized betas regarding the effect of climate-driven habitat suitability. The shape of the trajectory qualitatively matches expectations under PCM constraints. The standardized beta values for the effect of human population take a different shape. They are strongly negative for reindeer and reach their positive peak for red deer before strongly declining again, but neither of these trends are significant (threshold of *p* < 0.1). This suggests that anthropogenic resource depression did not drive changes in subsistence and thus the zooarchaeological record during this time.

## Discussion

The analyses presented in this paper suggest that changes in LUP foraging patterns, as seen in the zooarchaeological record of the period, were primarily climate-driven. Our results show that general changes in the composition of zooarchaeological assemblages throughout the LUP appear to be a product of climate-driven habitat shifts of prey species in almost all cases, not anthropogenic resource depression. Separating the effects of the two has been challenging for archaeologists^1,3,5,6^, yet by deploying a pSEM, we are able to account for the linkages between climate, human populations, and prey species abundances in the landscape specific for the LUP, and measure the impact that each of them have on zooarchaeological species composition. The results lead to the following observations which can be discussed in relation to our PCM-derived theoretical predictions.

High-ranked prey species should always be pursued upon encounter, regardless of their abundance, and their abundance in the archaeological record is therefore a measure of prey abundance on the landscape. If true, higher-ranked species will have better model fits, and this is reflected in the reindeer and boar data which shows better model fits than lower-ranked prey items such as red deer. The prediction is clearly reflected in the pSEM outputs which produce stronger beta values, smaller standard errors, and smaller *p*-values for the relationships between the habitat suitability and zooarchaeological abundances of reindeer and boar when compared to other species (cf. Fig, 3B). The pattern also extends beyond boar and reindeer to the other analyzed prey species as shown by the comparison of standardized beta values, where beta values decrease as standard error estimates increase as one descends in prey rank (cf. Fig. 4). This observation suggests that the presence and frequency of lower-ranked prey species in the zooarchaeological record is a result of a variable other than that species’ strict abundance on the landscape. Based on PCM, we expect the other variable to be the abundance of higher-ranked prey items, which is supported by our analysis of red deer zooarchaeological abundances relative to reindeer and boar zooarchaeological abundances (cf. Fig. 3C,D). This corroborates the hypothesis of constrained rationality, i.e., that the structure of the large mammal zooarchaeological record during the LUP is strongly shaped by cost–benefit regimes of human decision making and reflects the differential availability of high-ranked prey species in local environments.

When the local natural abundance of high-ranked prey species in the environment is greater, we expect high-ranked species to be proportionally represented in the zooarchaeological record, at least if their abundances are not simultaneously downregulated by human hunting pressure. This expectation is also broadly met given the results reported above, although with the caveat that we cannot independently control for absolute species abundances in each ecological context. Nonetheless, we observe that in the most suitable habitats for reindeer, LUP people take the most reindeer, and reindeer zooarchaeological frequencies in the worst reindeer habitats are lowest. The same is true for boar except for minor deviations in the poorest of boar habitats. A notable outlier to this pattern is the ABC red deer signal but this can be resolved by PCM-derived decision-making: As high-ranked prey animal species decline in abundance hunter-gatherers should incorporate more lower-ranked species into their diet in prey rank order. The incorporation of lower-ranked prey thereby not necessarily implies human-induced resource depletion but might similarly reflect changes in the species’ natural abundances. Our analysis of red deer zooarchaeological abundances relative to the higher-ranked species, reindeer and boar, support that in habitats less suitable for higher-ranked prey species, and thus have higher-ranked prey species in less abundance, hunter-gatherers incorporate lower-ranked resources in greater abundances. The finding is a clear example of diet breadth expansion^10^, however not as a product of human hunting pressure, but instead a product of environmental change.

The convergence between PCM-derived theoretical predictions and pSEM outputs confirms that on a macro-archaeological scale, pan-European trends in large mammal species compositions documented in the LUP zooarchaeological record are overwhelmingly shaped by local ecological conditions, reflecting the sorts of energetic and foraging return trade-offs documented in contemporary^75,86–91^ and prehistoric hunter-gatherer populations^5,73,92,93^. The changes noted in the structure of LUP faunal assemblages through time and space can thus primarily be linked to major climate shifts marking the end of the last glacial period with their associated effects on the distribution and composition of local environments and broader ecosystems^14–17^. This indicates a degree of adaptive plasticity among LUP populations in adjusting their foraging patterns to local conditions.

While human populations clearly impact species and habitats at greater population densities^28,94^ and have modified environments for thousands of years^95,96^, evidence for substantive super-regional impacts of human populations on animal abundances during the European Terminal Pleistocene is currently lacking. This suggests that population densities were not sufficiently high to depress faunal resources. It does not necessary mean that smaller scale, regional impacts are not discernible^9,97^, however, only that to detect these is outside of the scope of this study. Effective human population sizes during the LUP were relatively small when compared to later prehistoric or historic periods^98^. Such small population levels would in themselves reduce the potential ecological impact of human foragers on animal abundances, community structures, or broader ecosystem processes. Our results corroborate this view and show that in Europe there is no macro-archaeological evidence for population-induced changes in prey species abundances during the LUP. This provides a baseline against which earlier and later anthropogenic hunting impacts may be compared. It also raises the question of scale-dependence and conflicting observations, as earlier evidence for possible hunting pressure and lower-level human ecosystem impacts has been reported in the literature^99–102^, yet these findings are based on detailed osteological observations or isotope data, and their scales chiefly local. The seeming incommensurability of these with our findings may reflect different processes and especially analytical scales; ephemeral ecological impacts of Paleolithic foragers are likely to have occurred but are drowned out by larger-scale trends of climate change. Our results therefore do not call into question evidence for human ecological influences in the Pleistocene on local or even regional scales but rather caution that we cannot draw general conclusions from site-specific or regional observations.

A key limitation of this study is the discrepancy between the zooarchaeological and archaeological data. We here mitigate this shortcoming by agglomerating these data together by CTU. The result is a concatenation of the variation present within the archaeological record that may yet better help us understand the continuum of material and behavior across time and space. Better resolved zooarchaeological data with well-dated faunal assemblages would allow us to break away from relying on coarse archaeological attributions of CTUs and develop a better understanding of the spatiotemporal structure of variation observed within the zooarchaeological record. Further limitations include the potential underestimation of behavioral plasticity and attendant ecological preferences in animal species such as reindeer^103,104^, the extrapolation across vastly different ecosystem within Europe including but not limited to the Mammoth steppe in the north and east, and the likely impact of the changing availability and profitability of other resources, such as plants, fish, birds and other small game^56,105^. Unfortunately, marine resources, birds, and plants are not a part of our data set, and the small game component is only composed of marmots (*Marmota marmota*), hares (*Lepus* sp.), rabbits (*Oryctolagus cuniculis*), and beaver (*Castor fiber*). That said, in combination, these species make up a comparatively small part of the diet in the assemblages, ranging from an average of 6% for Mesolithic sites and less than 1% at Epigravettian and ABC sites^68^. As to whether the number of small game present in the data is real or a product of preservation or researcher bias is unknown, and recent studies have demonstrated the potential importance of the mass capture of small game resources to LUP hunters^106^, while others argue for the relevance of coastal or even marine resources accessible from the shore^107,108^.

In summary, we here assessed the impact of climate and human population changes on subsistence shifts as reflected in zooarchaeological records during the Late Upper Paleolithic in Europe. Building on the Prey Choice Model to derive the profitability of different prey species, a species distribution model to determine these prey species’ natural distributions, and a structural equation model to account for variable nonindependence, we generated a new analytical framework to quantify the relative contribution of climate change and human population pressure on the formation of the zooarchaeological record of LUP Europe. We find that regional changes recorded in zooarchaeological assemblages throughout the LUP are most likely a product of climate-driven species habitat shifts mediated through cost–benefit decisions of prey rank, rather than anthropogenic depression of key resources.

## Materials and methods

### Archaeological background

In general, Europe during the LUP was colder, wetter, and had greater seasonal variation than the Europe of the contemporary period^46,109–112^. In addition, climate over longer timescales was more volatile, punctuated by a series of glacial and interglacial periods (cf. Fig. 1). Climate regimes differed across the continent and Europe was generally characterized by a diversity of ecosystems. Even though the extension of these ecoclimatic contexts is subjected to dynamic re-adjustments over time, Northern Eurasia was broadly associated with the western extremity of the Mammoth steppe^113^, while the greater Mediterranean and Iberia formed distinct environmental contexts^114^. Situated at the westernmost fringes of the Mammoth steppe ecozone, Western France likely also harbored some unique ecoclimatic conditions^115,116^. For a detailed review of climate during the study period see^54,55^.

Archaeologically, the study period is characterized by a suite of hunter-gatherer techno-complexes and cultural units of varying chronological and spatial scope^46,58,117^, of which it is difficult to determine at which level these units are meaningful for human behavior^118–120^. To avoid this issue, we use a coarse-grain level of observation above traditional technocomplexes, resulting in six macro-archaeological units corresponding to broad differences in lithic techno-typology and referred to as Cultural Techno-Units (CTUs) throughout this paper. The Late Upper Paleolithic sensu stricto comprises two CTUs, the Epigravettian in Eastern Europe, south of the Alps and along the Mediterranean rim, and the Magdalenian north of the Alps and in Northwestern Europe^55,121–124^. Both complexes have traditionally been subdivided into Early, Middle, and Late phases, sometimes including a Final or Terminal phase overlapping with subsequent Final Paleolithic complexes. The subsequent Final Paleolithic is represented by the Arch-backed Complex (ABC)^55,124–128^, the Tanged Point Complex (TPC)^126,129–133^ and the very late Flat Blades and Bladelets Technocomplex (BBT; omitted here due to small sample size) dated to the Pleistocene-Holocene transition^56,134–137^. The Mesolithic, finally, synthesizes the plethora of earliest Holocene complexes documented and dated in the study region^138–141^.

Each of these macro-complexes is briefly described below.

#### Magdalenian

Includes all technocomplex designations labeled as Magdalenian including Final and Terminal Magdalenian facies as well as Magdalenian-descendant complexes such as the British Cresswellian^142^ and the Hamburgian^143^ of the North European Plain and Southern Scandinavia (including the latter’s later Havelte phase). The Magdalenian is generally characterized by invested laminar technologies with integrated or separate bladelet production systems^144^. Blade products mainly serve as blanks for domestic tools whereas bladelets are often backed and then used as inserts for modular tools and hunting equipment such as the spear thrower. Final Magdalenian complexes and Magdalenian-descendent complexes are distinguished by the emergence of special point types manufactured on blades such as the diagnostic shouldered points of the Hamburgian or the obliquely truncated Cresswell and Cheddar points of the Cresswellian. Reindeer and horse have been argued to be the main staple prey^122,145^, including ibex and snowy owl in mountain-near areas^146,147^. The Hamburgian has been hypothesized to index specialized reindeer economies^148^.

#### Epigravettian

Includes all technocomplex designations labeled as Epigravettian including regional variants such as the Romanellian of Italy^149,150^ and the Bouverian of northwestern Italy and southern France^151,152^. Similar to the Magdalenian, the Epigravettian is a complex characterized by invested laminar technologies with a generalized separation between blade-based domestic tools and bladelet-based implements^123,153^, although debate about its precise definition remain^154^. In contrast to the Magdalenian, however, Epigravettian variants are distinguished by backed blades and bladelets reminiscent of Gravettian character. The Late Epigravettian is often accompanied by geometric microlithic forms^155^. Prey animals include a variety of animals including boar, deer, bovids, and rugged terrain ungulates^156^.

#### ABC

Mainly comprises two technocomplexes: the Azilian of Western Europe and the so-called Federmessergroups (FMG) of Central and Eastern Europe^125^. Both technocomplexes are characterized by specialized bladelet technologies and a proclivity towards finely retouched as well as backed bladelets, often with a curved or arched edge. The ABC is often regarded to reflect woodland adaptations based on bow-and-arrow technologies^157^. The Azilian is currently interpreted to develop from the preceding Magdalenian^124^, whereas the status of the FMG and its relationship to the Azilian is contentious. The ABC seems to be associated with a broadening of human diets and mixed prey preferences, including species such as horse, reindeer and various woodland species^158^.

#### TPC

Includes the following technocomplexes: the Ahrensburgian and Epiahrensburgian of Northern, Central and Eastern Europe^130,133,159^, the Bromme complex of Southern Scandinavia^160,161^, following conservative assessments, and the Swiderian of East-Central and Eastern Europe^131,133,162,163^. The TPC is defined by the appearance of large, often robust and small tanged points accompanied by more or less developed blade technologies, some of which support highly invested bidirectional reduction systems. These points are characterized by pragmatic, often localized retouch and are highly variable in shape and size. TPC is mainly a phenomenon of the North European Plain and has traditionally been seen as an adaptation to high-mobility animals in open steppe landscapes such as reindeer, or thick-skinned species such as elk and perhaps beaver at the edges of boreal-tundra environments^46,157^.

#### (F)BBT

Mainly comprises two technocomplexes: the Belloisian or Long Blade Industries of Northern France^134,137^ and Britain and the Laborian of Western Europe^135^. This macro-unit is defined by its emphasis on blade production without large stemmed phenotypes, a focus on the broad face of laminar cores and resulting flat blade products, the use of large plain blades as knives and smaller retouched geometric implements^56,135,164^. BBT is associated with prey species such as boar, aurochs and deer and other species indicating the exploitation of closed landscapes^135^. BTT does not feature in the main analysis (see Archaeological background).

#### Mesolithic

Includes all technocomplex designations labeled as Mesolithic, early Mesolithic or Epipalaeolithic falling into the study period (up to 9k cal. BP) as well as regional variants such as the Beuronian of Southern Germany^138^, the Maglemose of Northern Germany, Britain and Southern Scandinavia^140^, the Sauveterrian of France^141^ and the Microlaminar Epipalaeolithic on the Iberian Peninsula^165^. The Mesolithic is generally characterized by the proliferation of microlithic technology, either bladelet-based or supported by incipient microburin technology. The main locus of variation pertains to the position and type of retouch and the attendant design choices as well as variation in geometric microlith forms. The Mesolithic is also accompanied by a surge of blade tools dedicated to woodworking. Mesolithic diets are chiefly broad spectrum and include a variety of species including deer, boar, aurochs, elk, beaver and sometimes reindeer^166,167^.

### Prey ranking and prey choice model

To begin to understand human foraging decisions, the prey species most important to LUP hunter-gatherers’ subsistence, and thus high-ranked, must be determined. To understand which prey species were high ranked, we need to (i) identify what large mammals were generally present in the ecosystems of the study period, (ii) estimate their (post-encounter) profitability, and iii) rank them according to their profitability.

Profitability is defined here within the PCM, as *post-encounter return rate* (PERR)^62,168–170^. Ranking prey species can be difficult, and in the past researchers have simply assumed that the mean body weight of a species corresponds with its ranking since body weight should roughly equal hunting returns^9,73–76^. While this may be true for some animal species, especially smaller animals easily captured, the approach is demonstrably not appropriate for larger animals as the relationship between body weight and profitability is non-linear^77–81^. To derive a more robust measure for PERR additional factors need to be incorporated, however. PERR is commonly defined as energy divided by handling costs (measured in time) to produce a rate of energy acquisition [LHS of Eq. (1)]. The standard prey choice decision-making equation then predicts that upon encounter a prey item is pursued/acquired if the prey item is within a given diet breadth. This is reflected in Eq. (1) wherein, *e* is energy, *λ* is prey density, and *h* is handling cost:

1 $$\frac{{e}_{j+1}}{{h}_{j+1}}>\frac{{\sum }_{i =1}^{j}{\uplambda }_{i}{e}_{i}}{1+{\sum }_{i=1}^{j}{\uplambda }_{i}{h}_{i}}$$

After ranking the *n* prey types by PERR, prey items are added to the equation in order of increasing rank until the condition of Eq. (1) is met. The highest *j* that satisfies Eq. (1) is the lowest-ranked prey type in the diet and thus defines the total breadth of the diet^62^. PCM assumes that if the condition of Eq. (1) is met, the forager takes the prey item. Guaranteed acquisition is an assumption meant to simplify the model and generally results in no issues, but when we talk about mobile game species, there is always the chance that a forager will fail to acquire a pursued prey item.

To incorporate the probability of pursuit failure into PERR, it is vital to (i) discount the energy gained, (ii) distinguish post-encounter pre-acquisition costs (e.g., pursuit costs) from post-acquisition costs (e.g., butchering, smoking, cooking, etc.), and (iii) discount the post-acquisition costs and energy gained by the probability of pursuit failure. In mathematical notation, this results in Eq. (2) wherein *e* is energy, *p* is the probability of failed pursuit, *h* is post-acquisition handling costs, and *c* is post-encounter pre-acquisition costs:

2 $${PERR}_{i}=\frac{{e}_{i}(1-{p}_{i})}{\left(1-{p}_{i}\right){h}_{i}+{c}_{i}}$$

### Prey rank analysis of LUP Europe

To understand what prey species were important to LUP foragers and may have structured their land-use, diet, and material culture, we first need to know the species that lived in LUP Europe. We draw our list of species from Pushkina and Raia^6^ (Supplementary Information 6)^82^. This table provides only bodyweight data, thus creating an issue of determining PERR. In order to overcome this obstacle, we draw on the African faunal data used in Lupo and Schmitt^79^ (Supplementary Information 5)^82^. While many of the African species are bovids, we assume that they are characteristically similar to European species, which are primarily cervids, in regards to the variables considered in PCM. We created a series of linear models to predict the parameters within our PERR equation [see Eq. (2)] for our LUP prey species based on weight (see Supplementary Information 1)^82^. While weight cannot accurately predict PERR or prey rank, it can predict accurately energy and handling costs. Using these predicted values for each LUP prey species, we then calculated each PERR.

Using the Lupo and Schmitt^79^ data, we created a series of linear models to predict the parameters necessary to determine PERR for the species list in Pushkina and Raia^6^. The linear models for each have varying levels of success. We can confidently estimate parameters *e* (*p* < 0.001) and *h* (*p* < 0.001) and to a lesser extent *c* (*p* = 0.085), but *p* (*p* = 0.225) is difficult to determine. That said, it appears that we often overestimate *c* and *p* for smaller prey species and underestimate them for larger prey species, meaning our parameters are conservative estimates for *c* and *p*. Estimates of prey rank and PERR are listed in Table 1. While many of these species have similar PERR (of which we are only able to estimate), with the top five species being within 1,000 kcals/hr, it is important to remember that this is a rate, and that the comparison of PERR is standardized by time, meaning that these differences increase as handling times increase.

### Archaeological site database

Sites dating to between the LGM and the Final Paleolithic were extracted from the Cologne CRC806 E1 database on European Late Upper Paleolithic/Magdalenian sites as originally published in Kretschmer^65^. The database was merged with the published information on direct radiocarbon dates obtained from the same sites also compiled by Kretschmer^65^. This integrated dataset was supplemented by sites listed in the CRC806 E1 database on the European Final Paleolithic^66^, again discarding all sites for which no direct dates could be obtained. All sites were checked for the availability of radiocarbon dates as recorded in the latest version of the Paleolithic Europe Radiocarbon Database (v28) curated by the University of Leuven^67^. The resulting list of dated sites in the study period with geospatial coordinates was then individually reclassified using the CTUs described above. Finally, sites and radiocarbon dates were added manually to CTUs for which the sample size was low by referring to the published and latest archaeological literature. All data is found in Supplementary Information 3^82^. To avoid double-counting of individual sites, we filtered them by spatial location and assigned century. Dates that originate from the same site and fall within the same century were removed from our dataset and the analysis (n = 2132; Fig. 1A).

### Zooarchaeological data

We draw in our zooarchaeological data from Boyle^68^. This dataset originally features relative proportions of prey species identified in zooarchaeological assemblages across Europe (n = 1258). Unfortunately, most of these data do not have associated spatial or temporal data, therefore we instead use their CTU association as the common denominator with which to pair them with the archaeological data. We re-classify the individual faunal observations contained in Boyle^68^ according to the higher-order archaeological units (provided as ‘Class3’ in the dataset) and remove all other entries. This results in a total sample of 804 observations (Supplementary Information 4)^82^.

Our analysis focuses on faunal resources, and while meat may have provided a significant portion of the calories for the LUP (if ethnographic observations of high latitude foragers and mean temperature are any indication of meat-dependence^71,171^), plants and small game may be more important for understanding the subsistence and diet breadth during this time. Being limited to faunal elements means that the archaeological record may have filtered out large portions of human behavior, particularly those associated with plant and marine foraging. Strictly focusing on animal resources may produce a biased perspective on subsistence and human lifeways, of which we need to be particularly aware.

### Species distribution modeling

Next, we selected the seven prey species with the highest PERR derived from the fitted parameters and created five species distribution models (SDM) for each species, excluding fallow deer (*Dama dama*) and ass (*Equus hydruntinus*). Fallow deer are not present in Europe during the LUP and the species of ass is only present in paleontological databases. To create each species’ SDM we used procedures outlined in the Wallace package^172^ and modern species data derived from GBiF^173^. The Wallace package uses a machine learning method, known as maximum entropy, to model presence points to pseudo-absence points, to determine the potential niche space of a species relative to the variables provided^174^.

Our SDMs used modern species data from the Northern Hemisphere and therefore used modern climate data from BioCLIM^175^. Our choice of variables is limited by the variables found in our paleoclimate data, so we selected mean temperature (bio01), low temperature (bio05), high temperature (bio06), and precipitation (bio12) as our variables for defining the climatic niche these species occupy at present. All SDM code are found in Supplementary Information 1^82^. All models are limited in their feature expansion to linear quadratic fits to avoid overfitting. Model regularization multipliers were selected based on mean and variation in AUC values produced from a four-fold cross validation (Table 2 in Supplementary Information 2)^82^. After creating the SDMs, we then hindcast the models over the CHELSA TraCE21k paleoclimate data. The CHELSA TraCE21k data are downscaled TRaCE21k data. The downscaling uses the CHELSA global climate model algorithm^63^. For our use, these data come in 100-year time slices from 22 to 9 kya using the same variables as our SDM (mean temperature, low temperature, high temperature, and precipitation). By using modern species data, we are assuming that the climate niches occupied by modern species adequately reflect the climate niches occupied by those same species during the terminal Pleistocene. While surely some shifts have occurred, particularly due to modern habitat destruction and impacts, we believe the climatological range represented in the modern data provides an adequate estimate of the range of habitats occupied by the different species. That said, our climate-based SDM models likely overestimate the habitat space of species, representing closer to a potential range rather than the realized range^176^.

Using our archaeological site database (n = 2465), which includes both spatial and temporal data, we then assigned each site (i) temporally to the century time slice of data for the CHELSA TraCE21k data using its median calibrated age rounded to the nearest century and (ii) spatially to a raster cell using its latitude/longitude. Next, we extracted the corresponding spatiotemporal cell value for the site. This creates the climate data for each site in our data. We then applied the SDM models over these climate values at our sites to determine how suitable each site is for each of the prey species.

### Summed probability distribution

To estimate regional population densities in Europe, we created a summed probability distribution (cf. Fig. 1b) P3K14C database ^64^. After downloading the database, we truncated the data to sites in Europe with radiocarbon dates ranging from 22kya to 9.1 kya. To account for multiple dates at the same site, we binned dates from the same site within 200 years and then applied a 200-year local mean smoother^177^. The resulting database provides relative ,population estimates throughout the LUP while avoiding potential edge effects^177,178^.

### The combined (archaeological) dataset

Last, we combined the datasets based on CTU association. We calculated the central tendency of each variable for each CTU. These include the central tendency of habitat suitability of each species for each CTU, the mean proportion of each species in the zooarchaeological record, the mean SPD value, and the mean maximum temperature of the warmest month (Table 1 in Supplementary Information 2)^82^. We used the central tendency because the zooarchaeological database lacks temporal and spatial data, and the archaeological data is missing zooarchaeological data. To overcome this issue, we distill the data to a compatible resolution, which is by CTU. This means that we are taking the central tendency of the habitat suitability of spatial and temporal positions for each CTU and pairing them with the mean zooarchaeological proportions of each species for each CTU. For a closer look at the variation and standard error of our estimates of these variables within the CTUs, see Supplementary Information 1^82^.

### Statistical analysis

We use a partial structural equation model (pSEM) to address issues of non-independence between our predictor variables^69^. The pSEM allows us to accurately assess the impacts of our variables on the zooarchaeological assemblage. Our model structure assumes that climate change effects both human populations and reindeer habitat suitability and thus reindeer populations, human populations impact reindeer populations, and that human populations and reindeer habitat suitability create the zooarchaeological record (Fig. 4A). Each model within the pSEM uses quasibinomial logistic regression.

Additionally, we recorded standardized betas and errors (Fig. 4B) produced by each pSEM model to test the assumption that the zooarchaeological record is structured by high-ranked prey species, with the prediction of declining importance in that species’ habitat as we decline in rank. Standardize betas represent the strength of a trend and the errors account for the uncertainty around that estimated trend.

## Data Availability

All data and code are available as supplementary information on Zenodo^82^.
